# A Retrotransposon Insertion in the 5′ Regulatory Domain of Ptf1a Results in Ectopic Gene Expression and Multiple Congenital Defects in Danforth's Short Tail Mouse

**DOI:** 10.1371/journal.pgen.1003206

**Published:** 2013-02-21

**Authors:** Francesca Lugani, Ripla Arora, Natalia Papeta, Ami Patel, Zongyu Zheng, Roel Sterken, Ruth A. Singer, Gianluca Caridi, Cathy Mendelsohn, Lori Sussel, Virginia E. Papaioannou, Ali G. Gharavi

**Affiliations:** 1 Department of Medicine, Columbia University College of Physicians and Surgeons, New York, New York, United States of America; 2 Department of Genetics and Development, Columbia University College of Physicians and Surgeons, New York, New York, United States of America; 3 Laboratory on Pathophysiology of Uremia, G. Gaslini Institute, Genoa, Italy; 4 Department of Urology, Columbia University College of Physicians and Surgeons, New York, New York, United States of America; The Jackson Laboratory, United States of America

## Abstract

Danforth's short tail mutant (*Sd*) mouse, first described in 1930, is a classic spontaneous mutant exhibiting defects of the axial skeleton, hindgut, and urogenital system. We used meiotic mapping in 1,497 segregants to localize the mutation to a 42.8-kb intergenic segment on chromosome 2. Resequencing of this region identified an 8.5-kb early retrotransposon (ETn) insertion within the highly conserved regulatory sequences upstream of *Pancreas Specific Transcription Factor, 1a* (*Ptf1a*). This mutation resulted in up to tenfold increased expression of *Ptf1a* as compared to wild-type embryos at E9.5 but no detectable changes in the expression levels of other neighboring genes. At E9.5, *Sd* mutants exhibit ectopic *Ptf1a* expression in embryonic progenitors of every organ that will manifest a developmental defect: the notochord, the hindgut, and the mesonephric ducts. Moreover, at E 8.5, *Sd* mutant mice exhibit ectopic *Ptf1a* expression in the lateral plate mesoderm, tail bud mesenchyme, and in the notochord, preceding the onset of visible defects such as notochord degeneration. The *Sd* heterozygote phenotype was not ameliorated by *Ptf1a* haploinsufficiency, further suggesting that the developmental defects result from ectopic expression of *Ptf1a*. These data identify disruption of the spatio-temporal pattern of *Ptf1a* expression as the unifying mechanism underlying the multiple congenital defects in Danforth's short tail mouse. This striking example of an enhancer mutation resulting in profound developmental defects suggests that disruption of conserved regulatory elements may also contribute to human malformation syndromes.

## Author Summary

Birth defects are a major cause of childhood morbidity and mortality. We studied the Danforth's short tail mouse, a classic mouse model of birth defects involving the skeleton, gut, and urinary system. We precisely localized the mutation responsible for these birth defects to a 42.8-kb segment on chromosome 2 and identified the mutation as an 8.5-kb transposon that disrupts highly conserved regulatory sequences upstream of the *Pancreas Specific Transcription Factor, 1a* (*Ptf1a*). The insertion disrupts a *Ptf1a* regulatory domain that is highly conserved across evolution and results in spatiotemporal defects in *Ptf1a* expression: we detected increased expression, temporally premature expression, and (most important for elucidating the mutant phenotype) the ectopic expression of *Ptf1a* in the notochord, hindgut, and mesonephros—the three sites that will give rise to organ defects in Danforth's short tail mouse. Our data also provide a striking example of how a noncoding, regulatory mutation can produce transient spatio-temporal dsyregulation of gene expression and result in profound developmental defects, highlighting the critical role of noncoding elements for coordinated gene expression in the vertebrate genome. Finally, these data provide novel insight into the role of *Ptf1a* in embryogenesis and lay the groundwork for elucidation of novel mechanisms underlying birth defects in humans.

## Introduction

Congenital malformations occur in about 3% of all live births and are a major cause of childhood morbidity and mortality [1], [2]. These defects can affect multiple systems, defining syndromes. Caudal regression syndrome (or caudal dysgenesis) is a major developmental syndrome characterized by malformation of the neural tube, caudal spine, the hindgut and lower limbs [3], [4]. Caudal regression can also occur with bilateral renal agenesis, and this form is usually fatal because it produces secondary pulmonary hypoplasia [3], [4]. Caudal regression most often occurs as sporadic disease; mutations in *HLXB9* cause Currarino syndrome (sacral agenesis, OMIM 176450), accounting for a minority of cases [5]. On the milder side of the clinical spectrum, isolated defects, such as renal hypoplasia or unilateral renal agenesis, are common but often remain subclinical [6], [7]. Kidney malformations are highly genetically heterogeneous. Mutations in the *PAX2* and *HNF1B* genes may account for up to 15–20% of pediatric renal hypoplasia [8]–[10]. However, the molecular basis for the majority of severe syndromes, such as bilateral renal agenesis and caudal regression are not well understood.

The *Danforth's short tail* mutant mouse is a classic Mendelian model of caudal malformations [11]. First described in 1930, this spontaneous mutation (symbol *Sd*) produces combined defects of the axial skeleton, urogenital system and distal gut [11], [12]. The homozygous mutant mice have fully penetrant abnormalities including truncation of the caudal vertebral column- resulting in a short or absent tail- as well as bilateral renal agenesis, colonic aganglionosis and absence of anorectal opening [12]. The *Sd/Sd* mice die shortly after birth due to these multiple organ malformations. The heterozygous (*Sd/+*) mutant mice exhibit short tails with complete penetrance and 30–40% incidence of unilateral renal agenesis; the solitary kidneys in the *Sd/+* mice are devoid of major structural defects and consequently, these mice have a normal lifespan [12], [13]. The *Sd* homozygote and heterozygote mutants thus represent excellent models of human caudal regression syndrome and isolated unilateral renal agenesis, respectively.

The earliest defect detected in *Sd/Sd* mice is the progressive disintegration of the notochord and the floor plate starting at E9.5, causing patterning defects in both neural tube and somites, leading to vertebral defects; the ureteric bud, derived from the mesonephric duct, either fails to reach or fails to induce the metanephric blastema, resulting in renal hypoplasia or aplasia; abnormalities in the development of the hindgut and cloaca result in the absence of an anorectal opening and could also be the cause of aganglionosis of the rectal pouch [12]–[16]. Studies of chimeric embryos have shown that the *Sd* cells are selectively lost from the notochord and ventral hindgut endoderm starting at E9.5, implicating a cell-autonomous defect in these tissues [17]; in rare instances that metanephric kidneys develop, however, *Sd* cells are robustly incorporated into chimeric kidneys, suggesting that the urogenital defect may be cell-nonautonomous or due to specific impairment of signaling for the mesonephric to metanephric transition [17]. More recent studies have shown that genetic ablation of the notochord with Diphtheria toxin recapitulates the axial defects observed in the *Sd* mutants, but notochord-ablated mice exhibited only kidney fusion and no noticeable defects in nephrogenesis, suggesting that an additional mechanism accounts for the renal agenesis in the *Sd/Sd* mouse [18]. Thus, identification of the genetic basis of the *Sd* mutation will provide insight into mechanisms of axial skeletal development and reconcile potentially contradictory findings about the origin of visceral defects in this mutant strain.

Previous studies have assigned the *Sd* locus to Chr. 2A3 but the mutation was not known [19]. Here we refined the *Sd* locus to a 42.8 kb interval and identified the *Sd* mutation as an insertion in the 5′ regulatory domain of *Pancreas Specific Transcription Factor 1a* gene (*Ptf1a*). This mutation results in ectopic expression of *Ptf1a* in the notochord, mesonephros and gut providing an explanation for the complete spectrum of abnormalities seen in *Danforth's short tail* mutants.

## Results

### Mapping of the *Sd* mutation to a 42.8-kb intergenic region on Chr. 2

The *Sd* mutation arose in 1930 on an outbred stock prior to generation of classical inbred strains. Mice carrying the *Sd* mutations were transferred in 1950 to Jackson laboratories in a linkage testing stock called E1, which was sequentially outcrossed to C57BL/6J, C3H/He and CBA and then maintained as closed colony until 1970 (Jackson laboratories). This colony, segregating the *Sd* mutation, was subsequently sibling mated at Jackson laboratories and named RSV/LeJ. We obtained RSV/LeJ-*Sd/+* mice and confirmed that *Sd/Sd*, *Sd/+* and *+/+* mice are obtained in an expected Mendelian ratio. RSV/LeJ-*Sd/Sd* mice were readily recognizable at birth because they manifested all the organ malformations defects originally described [12]–[14]. RSV/LeJ-*Sd/+* exhibited short tails with no other outwardly visible defects; on dissection, unilateral renal agenesis was detected in 10/28 (35%) of RSV/LeJ-*Sd/+* mice, consistent with previous reports [12]–[14]. RSV/LeJ- *+/+* exhibited long tails and were anatomically indistinguishable from other inbred strains.

**Figure 1:**
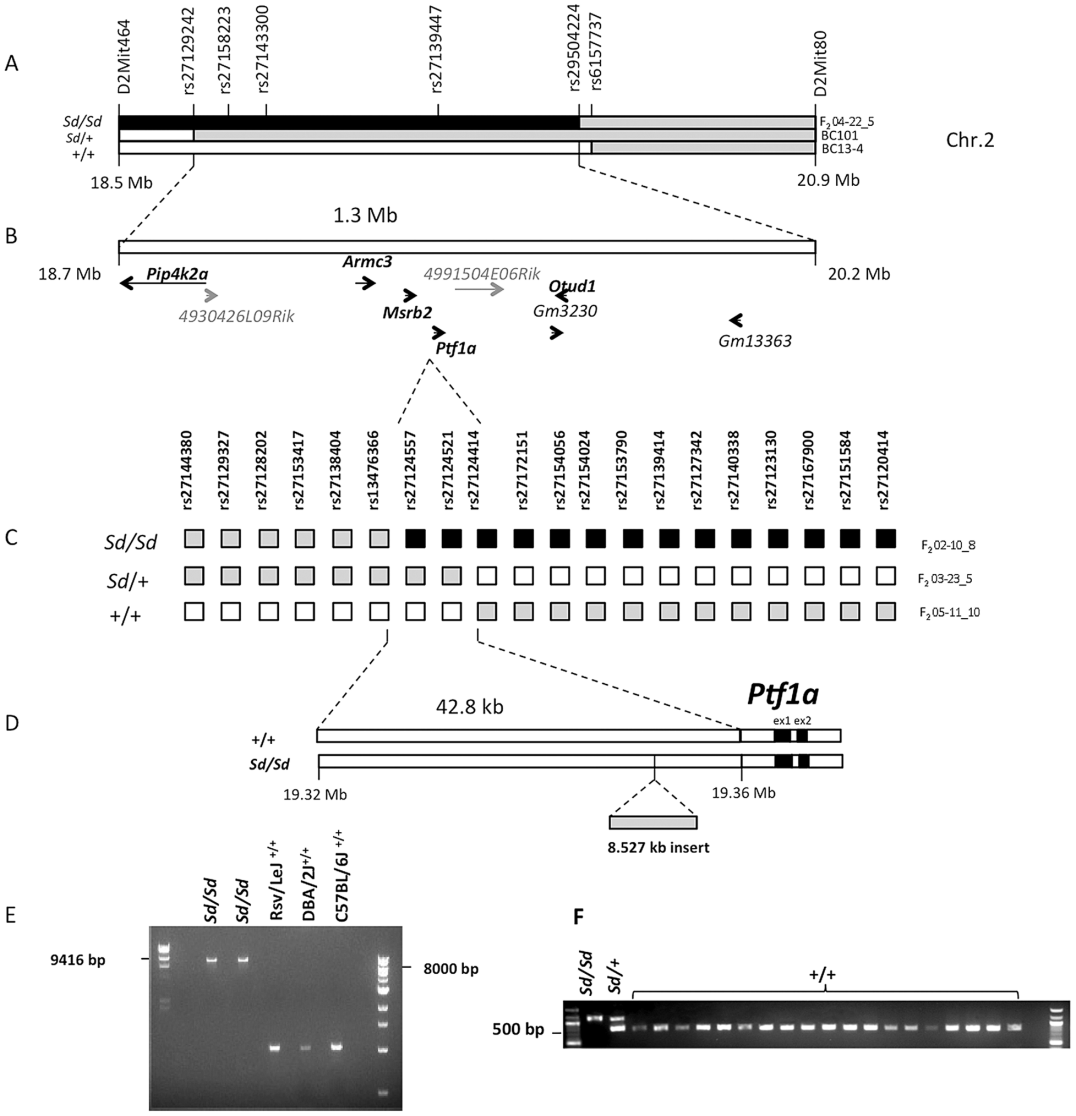
Fine mapping and identification of the *Sd* mutation. (A) Meiotic mapping on mouse Chr.2 on 174 backcross (BC) and 120 F2 intercross mice refines the *Sd* locus to a 1.3 Mb interval. The critical recombinants in 2 BC and 1 F2 mice are shown. The phenotype of each mouse (*Sd/Sd, Sd/+* and *+/+*) is indicated on the left. The genotypes are indicated by the color of each haplotype: black = RSV/RSV genotype; gray = RSV/B6 genotype; white = B6/B6 genotype. The location of genotyped markers is indicated above the haplotypes. (B) The initial mapping localized the *Sd* mutation to a 1.3 Mb interval on Chr. 2 between rs27129242 and rs29504224. This region contains 9 genes, of which five have mammalian orthologs (shown bold black). Predicted genes without mammalian orthologs are indicated in gray. (C) High resolution mapping in 1203 F2 mice. The critical recombinants in 3 F2 mice are shown. The phenotype and genotypes are indicated as in (A). (D) Localization of the *Sd* mutation to a 42.8 kb interval between rs13476366 and rs27124414, 12.2 kb upstream of the *Ptf1a* start site and identification of an insertion of 8.5 kb in the *Sd/Sd* mouse. (E) Long-range PCR identifies the insertion in 2 different mice *Sd/Sd* mutants but not in the RSV/LeJ strain and the two counterstrains used in the mapping study (C57BL/6J and DBA/2J). (F) Genotyping identified the insertion in *Sd/Sd*, and *Sd/+* samples but not in any additional mouse strain tested, including 4 wild-derived and 15 other laboratory inbred strains (listed in methods).

Previous studies had mapped the *Sd* locus to a 5 cM interval on Chr. 2 [19]. To confirm and refine this interval, we generated 174 backcross (BC) and 120 F2 intercross progeny (using C57BL/6J as the counterstrain). Analysis of linkage with 10 informative markers confirmed the mapping of the *Sd* locus to this region, yielding a peak lod score of 39 (p<6×10^−41^, Figure 1 and Figure S1). Three informative recombinants refined the locus to 1.3 Mb interval containing nine transcriptional units, including five genes with orthologs in other mammalian species (*Pip4k2a, Armc3, Mrsb2, Ptf1a, Otud1*, Figure 1A and 1B).

However, sequence analysis of exons and flanking regions as well as copy number analysis of all nine positional candidates did not identify any coding variants or intragenic copy number changes that distinguished RSV/LeJ-*Sd/Sd* mice from the background strain. Because the mapping was based on multiple critical recombinants in affected mice, this excluded the possibility of mismapping due to incomplete penetrance. Taken together these data indicated that the *Sd* mutation occurs in a noncoding region within this interval.

We therefore undertook further refinement of the *Sd* locus by meiotic mapping in additional F2 intercross mice. We did not identify informative markers between B6 and RSV/LeJ-*Sd/Sd*, or RSV/LeJ- +/+ across this minimal interval, suggesting that these strains share the same ancestral haplotype in this region. However, DBA/2J harbored informative SNPs and was therefore used as the counterstrain for fine mapping. We generated 1203 F2 intercross mice and genotyped 29 informative markers across the minimal 1.3 Mb interval. We identified 14 F2 progeny with recombinants within this interval, with 4, 7 and 3 mice exhibiting the wildtype, heterozygote and homozygote phenotypes, respectively. Among these, three critical recombinants localized the *Sd* locus to a 42.8 Kb intergenic region between rs13476366 and rs27124414, proximal to *Ptf1a* (Figure 1C, 1D). These mapping results were highly reliable because two of these critical recombinants occurred in affected mice exhibiting the homozygous and heterozygous mutant phenotype (Figure 1C).

### The *Sd* mutation is an insertion 12.2 kb upstream of the *Ptf1a* gene

We performed Sanger sequencing of the 42.8 Kb segment spanning the minimal meiotic interval in an RSV/LeJ-*Sd/Sd* and an RSV/LeJ-+/+ mouse, achieving 100% coverage of the interval with an average base call accuracy of 99%. We identified an 8.53 Kb insertion within this region in *Sd* homozygotes (located at nucleotide position 19,355,026 on Chr. 2, genome build 37.2). The insertion was present in homozygosity in RSV/LeJ-*Sd/Sd* mice, in heterozygosity in RSV/LeJ-*Sd/+* mice, and was absent in the background RSV/LeJ-*+/+* mice and in the two counterstrains used for the mapping study (DBA/2J, C57BL/6J) (Figure 1E). We also genotyped this insertion in a random sample of 142 mice from the F2 mapping progeny and found that it perfectly segregated with the heterozygote and homozygote *Sd* phenotypes. There were no other variants distinguishing RSV/LeJ-*Sd/Sd* from RSV/LeJ-*+/+* within the 42.8 kb minimal recombinant intervals, and the RSV/LeJ-*Sd/Sd* sequence otherwise shows identity with the C57BL/6J reference sequence (Figure 1F).

**Figure 2:**
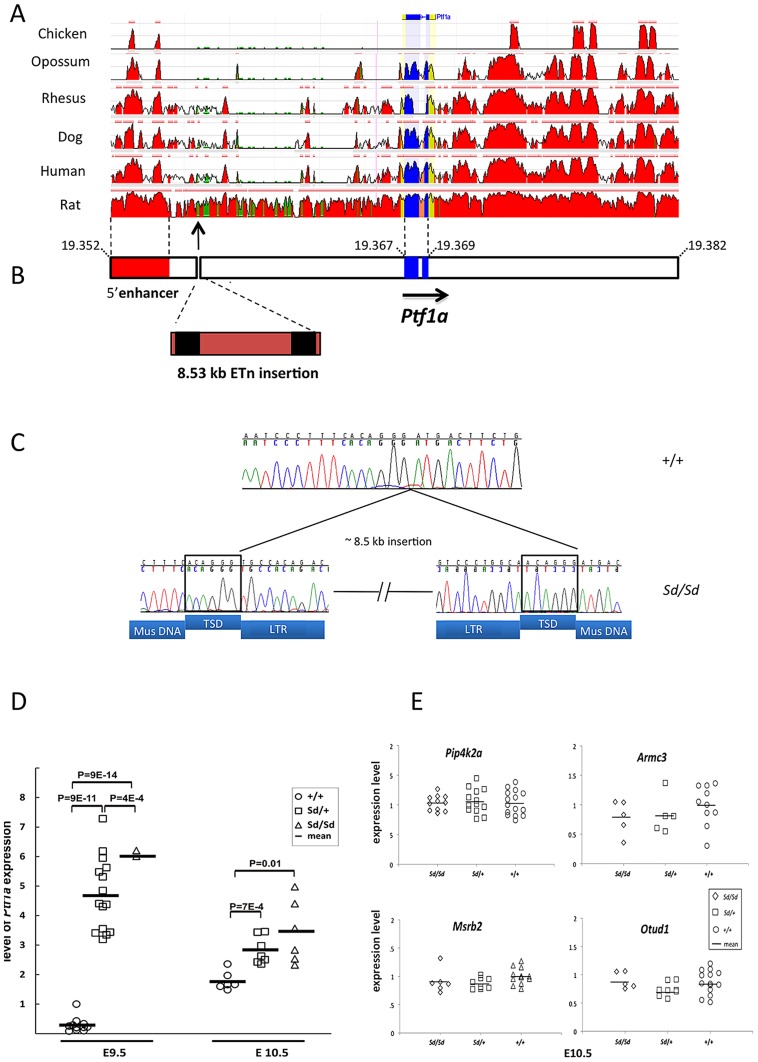
The *Sd* mutation is an insertion in the 5′ regulatory of *Ptf1a*. (A) Conservation of the *Ptf1a* genomic region across vertebrate species (ECR Browser at dcode.org). The height of the red peaks indicates the level of conservation. The *Ptf1a* exons are shown in blue. The *Sd* insertion is shown by the arrow. There is a highly conserved 5′ enhancer upstream of the Sd mutation (shown in red, located between positions 13.4–15.6 kb upstream of the *Ptf1a* start site). (B) Schematic representation of the *Ptf1a* locus. Sequence coordinates are shown in Mb (build 37.2). The *Ptf1a* exons are shown in blue. The location of the highly conserved 5′ enhancer element is shown in red. The *Sd* mutation is an insertion of an 8.527 kb ETn at nucleotide 19,355,020 on Chr. 2. The LTRs are indicated in black. The full sequence of the element is in Table 1. (C) Electropherogram of the site of the insertion in wild-type and *Sd/Sd* mutant mice. The target site duplications (TSD) are boxed. The sequences corresponding to LTRs, TSD and mouse genomic DNA (Mus DNA) are indicated below the nucleotide sequence. (D) Quantitative PCR of whole embryo shows up to 10-fold increased expression of *Ptf1a* at E9.5 in *Sd/Sd* mutants. *Ptf1a* expression remains significantly at E10.5. P-values for comparisons are indicated (t-test). (E) Quantitative PCR of whole embryo of neighboring genes with mammalian orthologs did not reveal any differences in expression between Sd mutants and wildtype mice (p>0.2 for all comparisons).

**Figure 3:**
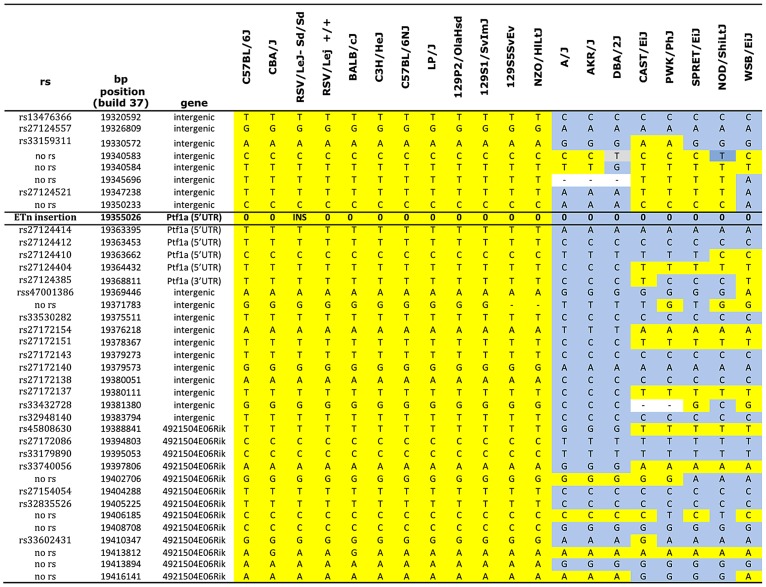
Haplotype map of the *Ptf1a* locus. Genotype data were obtained from the Mouse Phenome Database (Sanger data). All alleles identical to C57BL/6J are highlighted in yellow. Non- C57BL/6J alleles are highlighted in gray. The ETn insertion is indicated (INS). Missing genotypes are denoted by (-).

Sequence comparison with Repbase data indicated that the insertion is an endogenous retroviral element, with the closest alignment with the early retrotransposon (ETn) subtype (Figure 2A and 2B, Table S1). The insertion results in duplication of 6 bp at its flanking sites, without loss of reference sequence, which is typical of ETn sequences (Figure 2C) [20], [21]. Many copies of this mobile element are interspersed across the mouse genome, and transposition of ETn sequences into genic regions is responsible for many spontaneous mutant mouse phenotypes [20]–[23]. We further determined the haplotype on which this mutation occurred by genotyping 37 SNPs in the 95.5 kb interval surrounding *Ptf1a* (2.6 kb spacing, Table 1). This analysis demonstrated that in the RSV/LeJ-*Sd/Sd* and RSV/LeJ-*+/+* strains shared the same ancestral haplotype at the *Ptf1a* locus and therefore RSV/LeJ-*+/+* provides a valid reference haplotype for RSV/LeJ-*Sd/Sd*. This same haplotype is also present in ten other inbred strains (C57BL/6J, CBA/J, BALB/cJ, C3H/HeJ, C57BL/6NJ, LP/J, 129P2/OlaHsd, 129S1/SvImJ, 129S5SvEv, and NZO/HlLtJ, Figure 3). These data are consistent with the lack of polymorphism between C57BL/6J, RSV/LeJ-*Sd/Sd* and RSV/LeJ-*+/+* based on sequencing of the 42.8 kb minimal recombinant interval. Genotyping and Sanger Sequencing verified that the ETn insertion was absent in these ten strains as well as fifteen other strains with different haplotypes at the *Ptf1a* locus (Figure 1F). These data demonstrate that the ETn insertion is not an old polymorphism but is a new variant that arose on ancestral haplotype still commonly represented among laboratory inbred strains. Altogether, the precise mapping of the *Sd* locus to a 42.8 kb interval, the detection of an ETn insertion in the *Sd* mice and its absence in the RVS/LeJ background strain and twenty-five additional inbred strains, including strains with the same ancestral haplotype, demonstrated that we successfully identified the mutation responsible for the Danforth mouse phenotype.

**Table 1:** Phenotypes of (*Sd/+* X Ptf1a-cre/+) F1 mice.

*Genotype group*	*Ptf1a* Genotype	N	Kidney weight (g)	Kidney/Body Weight ratio	Kidney length (mm)	Tail length (mm)
***1***	*+/+*	13	0.11±0.03	0.008±0.0008	9.2±0.8	58.7±7.2
***2***	*Ptf1a-cre/+*	13	0.10±0.03	0.008±0.0008	8.6±1.2	56.6±14.4
***3***	*Sd/+*	11	0.09±0.03	0.009±0.001	7.9±1.1*	5.2±4.3#
***4***	*Sd/Ptf1a-cre*	13	0.09±0.02	0.009±0.001	7.9+0.8*	6.3±4.9#

The *Sd* mutation is indicated as a *Ptf1a* allele in column 2. Values are means ± standard deviations.

* P<0.003 versus group 1 (two-sided t-test).

# P<5×10^−8^ versus group 1 or vs. group 2 (two-sided t-test).

The ETn is inserted 12.2 kb upstream of the *Pancreas Specific Transcription Factor 1a* (*Ptf1a*) start site (Figure 2A). *Ptf1a* encodes a subunit of a trimeric Pancreas Specific Transcription Factor Complex (PTF1) which regulates cerebellar, retinal, pancreatic and spinal cord development [24]. The 5′ regulatory region of *Ptf1a* contains highly conserved elements, including a 2.3 kb autoregulatory enhancer domain located 13.4 kb upstream of the *Ptf1a* start site (Figure 2B), which normally directs *Ptf1a* expression to the dorsal spinal cord [25], [26]. Deletion of this 2.3 kb enhancer results in mislocalization of reporter constructs to the ventral spinal cord [26]. The *Sd* insertion occurs downstream of this highly conserved tissue enhancer, and displaces this element 8.5 kb upstream from its conserved position in the *Ptf1a* regulatory region (Figure 2A and 2B). The next closest gene *Mrsb2*, is located 64 kb proximal to the insertion, and has very low sequence conservation around its 3′ untranslated region. The data suggested that the ETn insertion is most likely to affect *Ptf1a* expression.

### The *Sd* mutation results in dysregulated *Ptf1a* expression

Analysis with GENSCAN predicted a few low probability open reading frames within the transposon sequence, but quantitative PCR analysis of whole embryo found no evidence that these sequences are transcribed, consistent with the known lack of transcriptional activity of ETn elements (data not shown) [20]. We examined expression of *Ptf1a* as well as other genes that were located 500,000 bp upstream or downstream of the *Sd* mutation. Quantitative PCR expression analysis of whole embryos at E9.5 and E10.5 revealed a consistent four-to ten fold increased expression of *Ptf1a* in *Sd* mutants (p<9×10^−14^ for comparison of *Sd/Sd vs. +/+* mice, Figure 2D). There were no detectable differences in expression of neighboring genes with mammalian orthologs at these two time points (*Pip4k2a*, *Armc3*, *OtuD1* and *Mrsb2*, Figure 2E). The other predicted genes in the region did not have mammalian orthologs and did not have detectable expression at these time points. These data rule out an effect on other genes located 500 kb upstream or downstream of *Ptf1a*.

**Figure 4:**
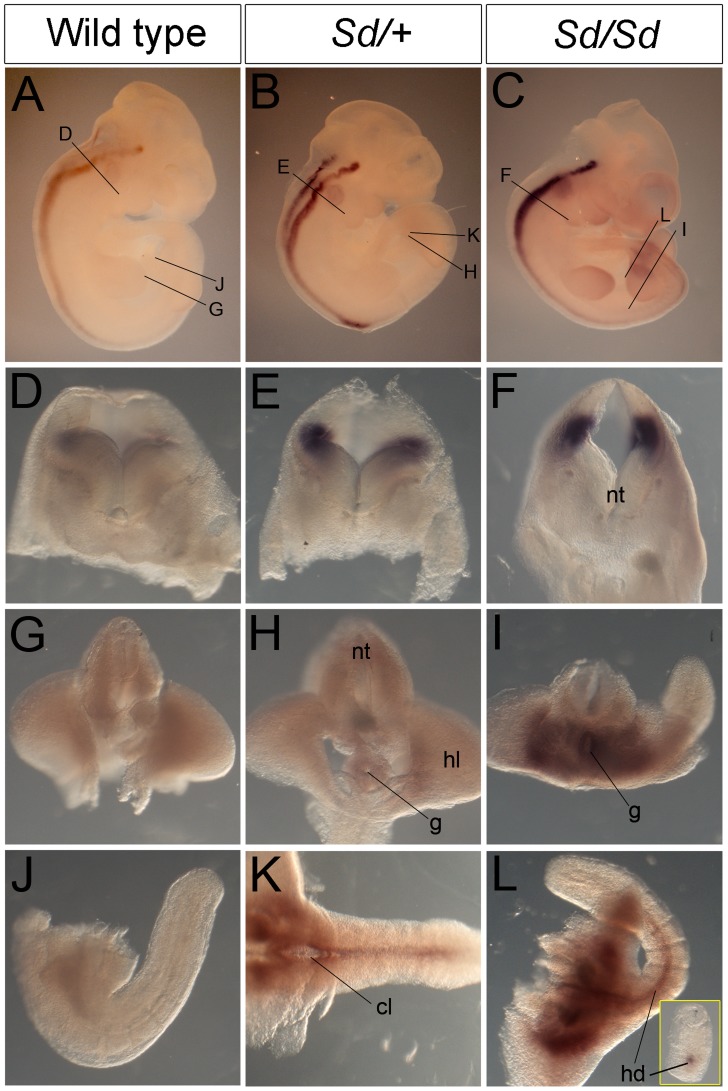
*Ptf1a* expression in wild-type, *Sd/+*, and *Sd/Sd* embryos at E10.5. (A–C) Whole mount ISH showing *Ptf1a* expression in wild type, *Sd/+* and *Sd/Sd*. embryos at E10.5. *Ptf1a* expression is seen in the hindbrain and throughout most of the neural tube. Lines indicate corresponding slices in D–I. (D–I) Slices from embryos in A–C showing *Ptf1a* expression at the level of hindbrain (D–F), hindlimb (G–I). (J–L) Isolated tails show ectopic expression of *Ptf1a* in the hindgut diverticulum (hd) in K,L. Inset in L is a slice close to the end of the tail showing expression in the hindgut diverticulum. Ectopic expression of *Ptf1a* is seen around the cloacal opening (cl) in K. nt-neural tube, g-gut, hl-hind limb.

To follow-up these findings, we performed in situ hybridization for *Ptf1a* in developing embryos. Consistent with a previous study [27], Ptf1a was robustly expressed throughout most of the length of the dorsal neural tube by E10.5 in wild type and was also seen in the same location in *Sd/+* and *Sd/Sd* embryos (Figure 4A–4F). In addition to this normal expression pattern, *Sd/+* and *Sd/Sd* embryos showed ectopic expression of *Ptf1a* in the hindgut, around the cloaca and in the hindgut diverticulum extending into the tail (Figure 4H, 4I, 4K, 4L). Consistent with E10.5 qPCR data, they're appeared to be a higher level of expression in the *Sd/Sd* embryos compared to *Sd/+* (Figure 4B, 4C, 4E, 4F), and also a higher level of endogenous expression in *Sd/+* compared to wild type (Figure 4A, 4B, 4D, 4E).

**Figure 5:**
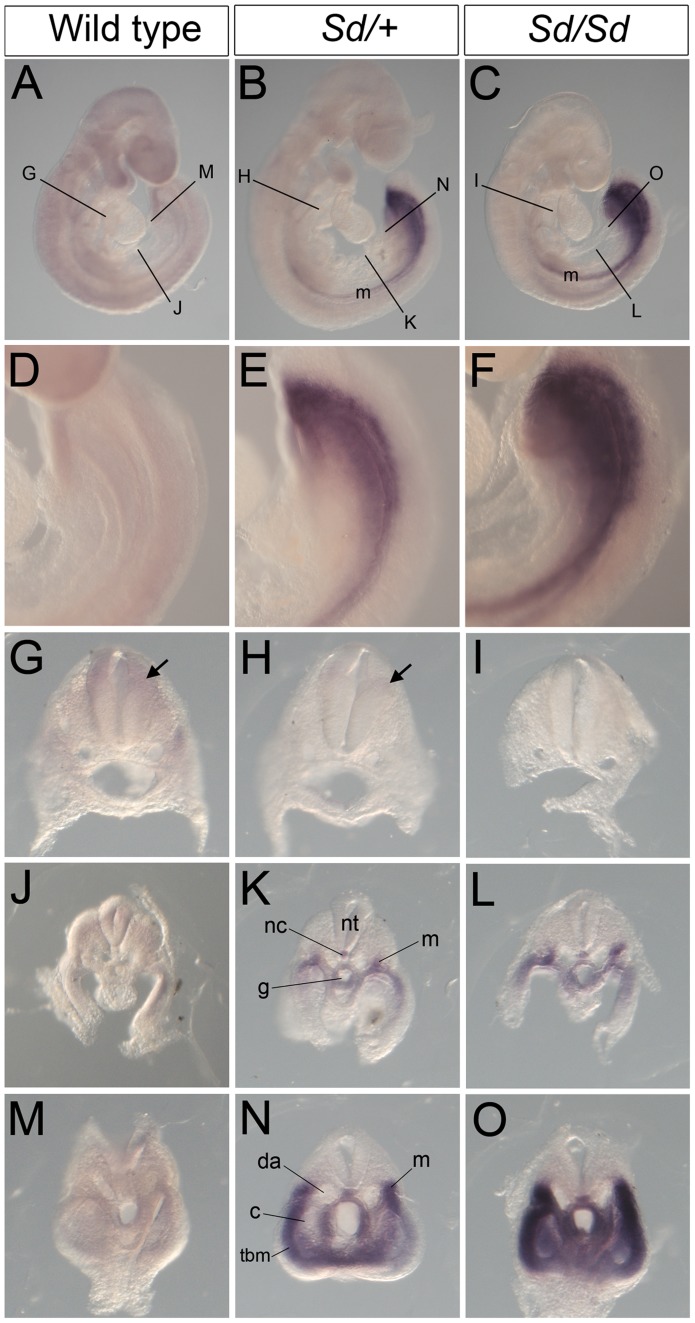
Ectopic expression of *Ptf1a* in *Sd/+* and *Sd/Sd* embryos at E9.5. (A–C) Whole mount ISH showing *Ptf1a* expression in wild type, *Sd/+* and *Sd/Sd* embryos at E9.5. Lines indicate corresponding slices in G–O. (D–F) High magnification views of the caudal end of the embryos shown in A,B,C. (G–O) Slices from embryos in A, B and C showing *Ptf1a* expression at the level of foregut (G–I), midgut (J–L) and the tail bud (M–O) of the embryo. *Ptf1a* is ectopically expressed in the posterior notochord (nc), mesonephros (m), and tail bud mesenchyme surrounding the gut tube (g) and the coelomic cavity (c) in the *Sd/+* and *Sd/Sd* embryos. da-dorsal aortae, nt-neural tube, tbm-tail bud mesenchyme. Arrows point to expression in the dorsal neural tube.

**Figure 6:**
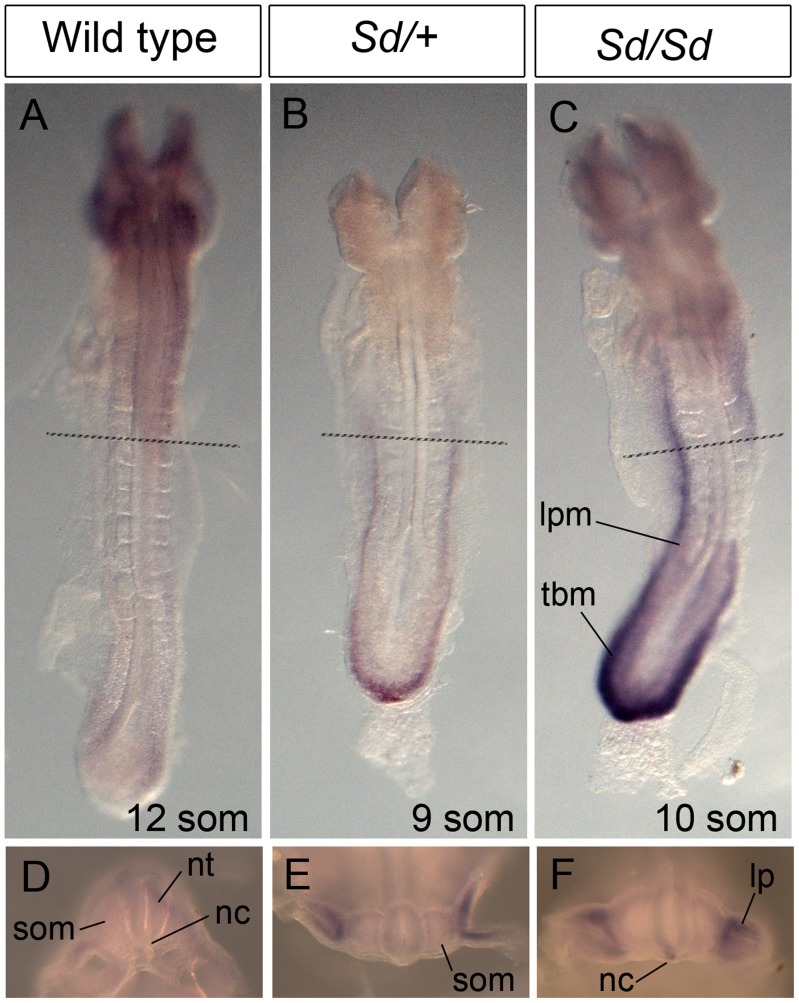
Ectopic expression of *Ptf1a* in *Sd/+* and *Sd/Sd* embryos at E8.5. (A–C) Dorsal view of whole mount ISH showing *Ptf1a* expression in wild type, *Sd/+* and *Sd/Sd* embryos at E8.5. Lines indicate corresponding slices in D–F. (D–F) Slices from embryos in A–C at the level of the fourth somite. *Ptf1a* is ectopically expressed in the lateral plate mesoderm (lpm) and tail bud mesenchyme (tbm) of *Sd/+* and *Sd/Sd* embryos and in the notochord (nc) of *Sd/Sd* embryos. nt-neural tube, som-somite.

A day earlier at E9.5, extensive ectopic *Ptf1a* expression was evident in *Sd/+* and *Sd/Sd* embryos in the tailbud mesenchyme (Figure 5B, 5C, 5E, 5F, 5N, 5O), the notochord and hindgut (Figure 5K, 5L, 5N, 5O), and throughout the length of the developing mesonephros and mesonephric duct (Figure 5B, 5C, 5K, 5L). Ectopic expression was evident even at E8.5 in the lateral plate mesoderm and tail bud mesenchyme of *Sd/+* and *Sd/Sd* embryos (Figure 6A, 6B, 6C, 6E, 6F) and the notochord of *Sd/Sd* embryos (Figure 6C, 6F). In summary, we detected ectopic *Ptf1a* expression in every organ that will manifest a developmental defect in *Sd/+* and *Sd/Sd* mice - the notochord, the mesonephros and the hindgut.

These data suggested that dysregulated timing and localization of *Ptf1a* expression is responsible for the Danforth phenotype. Nonetheless, because increased *Ptf1a* expression was also detected in mutant embryos, we attempted to distinguish hypermorphic from neomorphic effects of the *Sd* allele by crossbreeding *Sd/+* mice to *Ptf1a* haploinsufficient mice (*Ptf1a-cre* mice, reference [28]). If the Danforth phenotype was solely due to increased expression of *Ptf1a*, then reduction of *Ptf1a* gene dosage should attenuate the organ malformations. While the *Sd* mutants had significantly reduced kidney and tail lengths, there were no phenotypic differences between *Sd/+*, and *Sd/Ptf1a-cre ^mice^* at weaning (Table 1). Histologic analysis of the kidneys also did not reveal morphological defects, consistent with prior reports of normal histology in *Sd* heterozygotes [12]–[16]. The absence of rescue by *Ptf1a* haploinsufficiency suggests that inactivation of one wild-type allele could not compensate for the increased expression from the *Sd* allele or more likely, that the neomorphic effects of the *Sd* allele predominate in the pathogenesis of malformations in the Danforth mice.

Only a few PTF1a targets are known. Recent data suggest that PTF1a regulates *Mnx1*, *Nkx6-1*, *Bmp7*, *Dll1* and *Onecut1* expression [29]. However, we did not detect any differences in expression of these genes between *Sd/+, Sd/Sd* and *+/+* mice at E 9.5 (Figure S2), suggesting that increased *Ptf1a* expression is not sufficient to activate these particular targets in this tissue context and this time point.

## Discussion

Danforth first described his spontaneous mutant strain over 80 years ago [11]. Since that time, this strain has been studied as a classical model of developmental defects of the spinal cord, hindgut and the urogenital tract [12]–[19]. Although the *Sd* locus was assigned to Chr. 2A3 in 1980, the underlying mutation had not been identified. We used meiotic mapping in 1203 F2 mice to precisely map the mutation to a 42.8 kb intergenic region. This segment contained an ETn insertion upstream of *Ptf1a* in the mutant strain, which was absent in the RSV/LeJ background strain and twenty-five wild-derived or classical inbred strains. SNP analysis of the *Ptf1a* locus suggests that the mutation arose on an ancestral haplotype that is shared by at least eleven laboratory strains, including RSV/LeJ and three strains used in the derivation of this laboratory strain (C57BL/6J, CBA/J and C3H/HeJ). Our findings are independently supported by accompanying papers (by Vlangos et al. and Semba et al.) who used a alternatives approaches to identify the same mutation. Taken together, these data establish that we have identified the genetic basis of the *Sd* mutation. The presence of a noncoding mutation explains the difficulties in identifying the genetic lesion in the Danforth strain since its initial description in 1930. We observed perfect co-segregation of all of the skeletal, urogenital and gastroenterological phenotypes in all affected F2 and BC mice generated in this study, indicating that the noncoding mutation is at the origin of the entire spectrum of defects. The ETn insertion occurs within the regulatory sequences upstream of *Ptf1a*, and is associated with dysregulated dosage, timing and localization of expression. These data implicate dysregulated *Ptf1a* expression as the cause of developmental defects in the Danforth mouse and indicate that *Sd* should be recognized as a neomorphic *Ptf1a* allele.

*Ptf1a* is a member of the trimeric transcriptional complex PTF1, and loss of function mutations result in recessive cerebellar and pancreatic hypoplasia in humans and mice [24]–[26], [30]–[34]. *Ptf1a* has highly restricted temporo-spatial expression during embryogenesis [24]–[26], [30]–[33]. Transient *Ptf1a* expression between E10 to E13 initiates the genetic programs required for specification of dorsal horn neurons, the cerebellum and the pancreas; *Ptf1a* expression subsequently declines to undetectable levels, with postnatal expression present only in pancreatic acinar cells [24], [31], [32], [35]–[42]. This tightly regulated temporal and spatial expression pattern is controlled by highly conserved regulatory elements upstream and downstream of *Ptf1a* but the consequences of regulatory mutations have not been reported. The *Sd* mutation occurs in the vicinity of a highly conserved enhancer sequence (positioned from 13.4 to 15.6 kb upstream of the *Ptf1a* start site), which contains multiple autoregulatory domains required for restricting expression to the dorsal spinal cord and maintaining expression in the adult acinar cells (Figure 2B) [25], [26]. Reporter constructs lacking this enhancer are ectopically expressed in the ventral spinal cord [26] and although not specifically reported, are also evident in the hindgut at E10.5 (see reference [26], Figure 1B), consistent with the mislocalized expression pattern in *Sd/+* and *Sd/Sd* mutants (Figure 3, Figure 4). The retrotransposon insertion may disrupt this enhancer or a neighboring negative cis-acting element, or may act as a broadly-acting positive regulator, resulting in ectopic expression of *Ptf1a* in *Sd* mutants.

We show that each tissue that has been implicated as being primarily affected in *Sd* mutant mice – the notochord, the mesonephric duct and the hindgut – shows ectopic *Ptf1a* expression at a critical stage in its early development, indicating that dysregulated *Ptf1a* expression is at the origin of the developmental defects arising from these compartments. Notably, ectopic expression of *Ptf1a* is present in the notochord at E8.5, prior to or coincident with the earliest notochord defects and prior to the start of notochord disintegration [12]–[16]. Our data also suggest that the mouse embryo is very sensitive to *Ptf1a* gene dosage as *Sd/Sd* mice, which have a higher level of ectopic expression, consistently manifest more severe axial defects and near complete penetrance of bilateral renal agenesis resulting in death, whereas *Sd/+* animals have less severe defects compatible with survival.

PTF1a requires two cofactors to form an active transcriptional complex [34], [37]. If its canonical partners were present, its misexpression may activate its direct downstream targets, ectopically initiating neural or pancreatic developmental programs [43]. Alternatively, PTF1a may form transcriptional complexes inappropriately in regions where it is ectopically expressed, interfering with normal developmental processes and impairing notochord, urogenital and hindgut development. At present, the downstream targets of *Ptf1a* have not been comprehensively identified. We examined five known *Ptf1a* targets but did not detect increased expression in *Sd* mutant mice, indicating that unbiased genome-wide approaches will be required to discover dysregulated developmental programs downstream of ectopic *Ptf1a* expression.

The present findings provide a unifying mechanism for the multiple developmental defects in the Danforth mouse and reconcile several prior observations. For example, our data confirm a previous study that used genetic interaction to infer that *Sd* is a gain-in-function mutation [44]. The present data also explain why the Danforth mutation was not fully recapitulated by ablation of the notochord [18] and could not be explained based on dysregulation of any single embryonic compartment [17], as ectopic *Ptf1a* expression likely misdirects developmental programs independently in each affected tissue. Now that we identified the initiating genetic lesion, future studies can determine the precise molecular cascade leading to the developmental defects in each compartment.

To date, the vast majority of developmental defects reported in mice and humans are produced by coding mutations that result in loss of function of the encoded protein; developmental defects arising from disruption of conserved regulatory elements have been less frequently described [45]. Comparative genomics studies of bony vertebrates have identified highly conserved sequences that are enriched around genes that have tissue-specific enhancer activity, acting as developmental regulators [45]–[47]. The *Sd* mutation provides a striking example of a cis-regulatory mutation that produces profound developmental defects that are quite distinct from phenotypes resulting from simple loss/gain of function mutations. The Danforth mouse can thus serve as an excellent model for dissecting the role of enhancer elements on the temporo-spatial regulation of gene expression in vertebrate development. These data also suggest that mutations in conserved regulatory elements may contribute to human malformation syndromes. For example, we recently studied 522 children with kidney malformations and identified 72 rare copy number disorders that disrupt coding segments, accounting for up to 16.6% of cases [48]. However, we also identified many rare or unique intergenic CNVs in this population, suggesting that disruption of noncoding elements may also play a pathogenic role in this phenotype [48]. Given the high sequence conservation in the *Ptf1a* 5′ regulatory region, this segment is a good candidate for mutational screening in larger patient populations with caudal regression, axial or urogenital defects.

## Methods

### Mice

The Association for Assessment and Accreditation of Laboratory Animal Care guidelines were followed for all animal procedures, and all procedures were approved by the Institutional Animal Care and Use Committee of Columbia University. All inbred strains including the strain carrying the *Sd* mutant allele (RSV/LeJ-*Sd/+*) mice were purchased from The Jackson Laboratory. To refine the *Sd* locus, we generated 174 backcross and 120 intercross mice between RSV/LeJ-*Sd/+* mice and C57BL/6J mice and also a second mapping F2 intercross cohort of 1203 mice by intercrossing (RSV/LeJ-*Sd/+* x DBA/2J) F1 mice. Mice were phenotyped at birth by visual inspection. Affection status was assigned based on presence of the short tail in homozygotes and heterozygotes. While heterozygotes have no other outwardly visible phenotypes, the homozygote mutants are readily recognizable based on the presence of caudal agenesis, and on dissection have major gut and urogenital malformations as previously described [11], [13], [14], [16]–[19]. The Ptf1atm1(cre)Wri or Ptf1a–cre mice (carrying a Cre recombinase replacing the *Ptf1a* protein-coding region, resulting in a *Ptf1a* null allele) were obtained from Wright and colleagues [28]. To examine the effect of *Ptf1a* haploinsufficiency on the heterozygote phenotypes, we generated F1 hybrids between *Ptf1a–cre/+*mice and RSV/LeJ-*Sd/+* mice. Phenotypes were determined at weaning (postnatal day 22), by measurement of tail length, kidney length and kidney weight. H&E staining of kidneys were also performed. Genomic DNA was isolated using the Genomic DNA Isolation kit (Lamda Biotech).

### Genotyping and analysis of linkage

Marker loci for inbred strains were obtained from the Mouse Phenome Database (http://phenome.jax.org/); we filtered these for SNPs that were polymorphic between C57Bl/6J, DBA/2J and RSV/LeJ. The BC group was genotyped using microsatellite markers across the *Sd* locus. Multipoint lod score was calculated using the R/QTL package, utilizing the discrete trait analytic model. The F_2_ cohort was genotyped with 29 informative SNPs distributed across the 1.3 cM region delimited by rs27129240 and rs29504224 (Sequenom Mass Array system, Columbia University Genotyping facility). To define the haplotype at the *Ptf1a* locus, we searched SNPs in the Mouse Phenome Database (MPD) and genotyped 37 SNPs that differentiated common haplotypes between 17 inbred strains sequenced by the Sanger Center [49]. Haplotype analysis was performed by Sanger sequencing of RSV/LeJ-+/+, RSV/LeJ-*Sd/Sd*, C57BL/6J and DBA/2J The genotypes in C57BL/6J and DBA/2J mice demonstrated a 100% concordance with genotypes from dbSNP, confirming accuracy of Sanger sequencing.

### Sequencing

Gene annotation was performed using NCBI database (http://www.ncbi.nlm.nih.gov/) and UC Santa Cruz genome browser (http://genome.ucsc.edu/). Mutational screening of the positional candidate genes was performed by Sanger sequencing of exons and flanking introns, comparing RSV/LeJ-*Sd/Sd* mouse to a RSV/LeJ- *+/+* mouse. Long range-PCR was performed in order to amplify the insertion in the *Sd/Sd* mutant (TaKaRa long range PCR). We performed Sanger sequencing of the 42.8 kb minimal recombinant interval in an RSV/LeJ -*Sd/Sd* and RSV/LeJ *^−^+/+* mouse. We achieved 100% coverage of this interval with an average base call accuracy of 99% (Phred scores of 20); there were no gaps or ambiguity in the interval. Bidirectional sequencing was obtained for 76% of the region. The sequence of the insertion was analyzed by alignment with the Repbase data, the most commonly used database of repetitive DNA elements (http://www.girinst.org/repbase/index.html ref [50]). From The Jackson Laboratory, we obtained genomic DNA from 25 strains of mice which comprehensively represent all Mus musculus sub-species, including 4 wild-derived (PWD/PhJ, MOLF/EiJ, WSB/EiJ, CAST/EiJ) and 21 inbred strains (DBA/2J, DBA/1J, C57BL/6J, C57BL/6NJ, BALB/cJ, FVB/NJ, RIIIS/J, C3H/HeJ, AKR/J, NOD/LtJ, SJL/J, 129/SvimJ, 129/SvEv, 129P2/OlaHsd, CBA/J, CFW, SWR/J, BTBR T<+>tfJ, NZW/LacJ, KK/HlJ, and A/J).

### Quantitative analysis of cDNA

Timed embryos were collected at embryonic day (E) 9.5, 10.5 and E12.5 (where E0.5 is the day of detection of the vaginal plug). Comparisons were made between littermates with differing genotypes. RNA was isolated from whole embryos with TRIzol (Invitrogen) followed by DNaseI treatment and clean-up using the RNeasy mini kit (Qiagen). cDNA was generated with the Omni-Script kit (Qiagen). cDNA levels were quantified in duplicate by qPCR using SYBR Green Mix on an IQ thermal cycler (Bio-Rad). The same internal control was included in each run to standardize each qPCR run, and β-actin was used as reference gene (using Pfaffl algorithm). Expression levels were standardized to the same E10.5 wild-type mouse embryo.

### Embryo dissections and in situ hybridization

Embryos were collected at E8.5, E9.5 and E10.5 and yolk sacs were removed for PCR genotyping. Embryos were fixed in 4% paraformaldehyde (PFA) (Fisher), washed in PBT, dehydrated in methanol and processed for whole mount in situ hybridization (ISH) as described previously [51]. Samples at each developmental stage were processed together and images captured under identical settings to allow qualitative comparison of staining intensity between genotypes. The *Ptf1a* probe was generated by cloning the full-length *Ptf1a* cDNA from mouse embryonic pancreas into the TOPO blunt PCR cloning vector (Invitrogen). Briefly, embryos were bleached in hydrogen peroxide for an hour, treated with proteinase K (10 µg/ml) (Roche) and fixed in PFA. Further the embryos were incubated at 65°C in hybridization buffer for an hour and then digoxygenin labeled *Ptf1a* antisense RNA probe was added to hybridization buffer overnight. The following day the embryos were washed with solutions of decreasing stringency of saline sodium citrate salt solutions, washed in Tris-buffered saline with tween (TBST) and incubated in alkaline-phosphatase labeled anti-digoxygenin (Roche) in TBST overnight at 4°C. On the third day the embryos were washed in TBST followed by an overnight wash. On day four the embryos were developed using the BM purple (Roche) solution in dark. The embryos were washed in PBT after developing, post fixed with PFA and stored in PBT until photographed on a Nikon SMZ1500 dissecting microscope.

## Supporting Information

Figure S1 Multipoint lod score map of the *Sd* locus.
(PDF)

Figure S2 Quantitative PCR of PTF1a targets in whole embryo at E9.5.
(JPG)

Table S1 Annotated sequence of the ETn insertion at the *Sd* locus.
(PDF)
