# Supplementary material for: A Retrotransposon Insertion in the 5′ Regulatory Domain of Ptf1a Results in Ectopic Gene Expression and Multiple Congenital Defects in Danforth's Short Tail Mouse
Source: PLoS Genet. 2013 Feb 21;9(2):e1003206. doi: 10.1371/journal.pgen.1003206 (PMC3578747; doi:10.1371/journal.pgen.1003206)
Supplement: Figure S1 — Multipoint lod score map of the Sd locus. (PDF) [file pgen.1003206.s001.pdf]

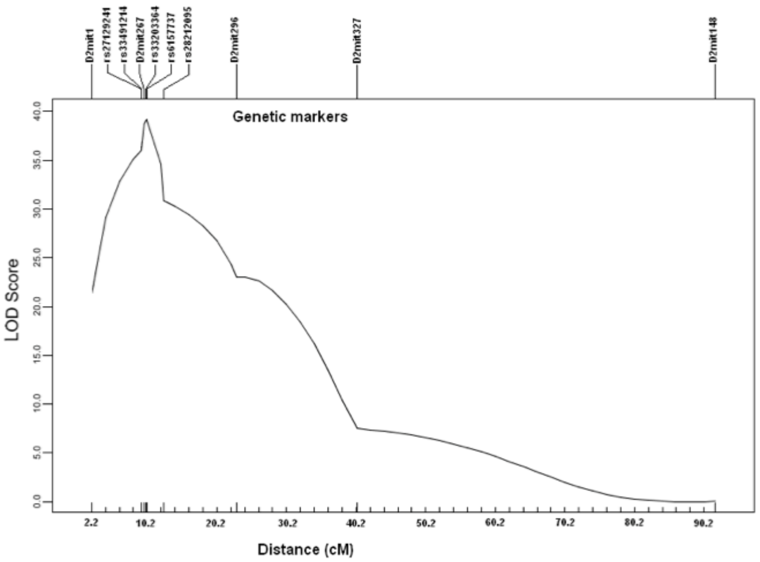

Supplementary Figure S1. Multipoint lod score maps the *Sd* locus to a 1.3 Mb region on mouse chromosome 2 Genetic distance on mouse Chromosome 2 is indicate on the X-axis (cM). the Y-axis shows the multipoint lod scores.
