# Supplementary figures and images for: A Retrotransposon Insertion in the 5′ Regulatory Domain of Ptf1a Results in Ectopic Gene Expression and Multiple Congenital Defects in Danforth's Short Tail Mouse
Source: PLoS Genet. 2013 Feb 21;9(2):e1003206. doi: 10.1371/journal.pgen.1003206 (PMC3578747; doi:10.1371/journal.pgen.1003206)

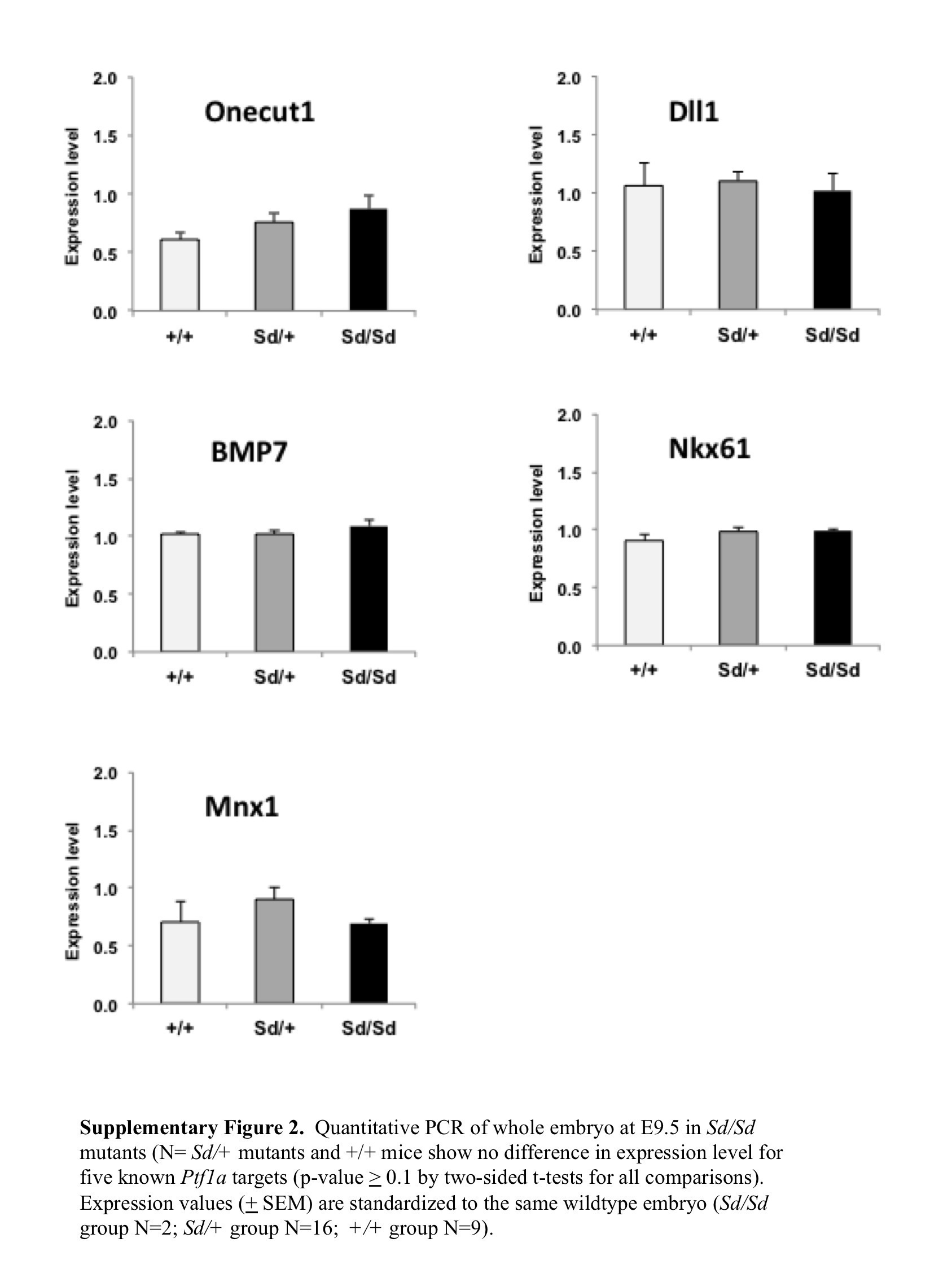

Supplement: Figure S2 — Quantitative PCR of PTF1a targets in whole embryo at E9.5. (JPG) [file pgen.1003206.s002.jpg]
