# Supplementary material for: A Retrotransposon Insertion in the 5′ Regulatory Domain of Ptf1a Results in Ectopic Gene Expression and Multiple Congenital Defects in Danforth's Short Tail Mouse
Source: PLoS Genet. 2013 Feb 21;9(2):e1003206. doi: 10.1371/journal.pgen.1003206 (PMC3578747; doi:10.1371/journal.pgen.1003206)
Supplement: Table S1 — Annotated sequence of the ETn insertion at the Sd locus. (PDF) [file pgen.1003206.s003.pdf]

Supplementary Table 1. Sequence of the ETn insertion at the *Sd* locus. The wildtype flanking mouse sequence is indicated in red. The target site duplication is shown in blue. The RepBase alignments are shown below the table. The LTRs are indicated in bold (bp 60-908, 7739-8585). The segment aligning to ETnERV3 - ERV2 Endogenous Retrovirus is shown in green (bp 915-6301). The segment aligning to an internal portion of ERV2 Endogenous Retrovirus (bp 6302-7738) is shown in purple. The transposon sequence was aligned with Repbase reference data. (http://www.girinst.org/repbase/index.html)

From	To	Repbase Sequence	From	To	Sim	Pos/Mm:Ts	Score
60	908	RLTR13A	4	857	0.9301	1.4062	6024
915	6301	ETnERV3	186	5797	0.9629	1.3828	42993
6302	7738	ERVB2_1A-I_MM	1	1435	0.9798	1.3333	13065
7739	8585	RLTR13A	6	857	0.9299	1.4062	6008

GCACAGACACTGTGGTCCACACTGTCTCATTGCCACTTTGGCTAATCCCTTTCACAGGG**TGCCACAGACAATCTGGG**
TCCCTGGTCTGTGAAGAACGGGATTTCTTAGGCAGATGGACAAAGATTCCGGATGAAAATGACAGACAGATCA
CACGAGAAATGTGTTGAATCTGAATGTATTGTTCTGGTTGAGCTTCAGACTTATATAACAACGAATTATCAGA
GGATACAAATCACAAAAAGACAAGATACACTGAAATTCACCAGTTACAGCAGAAAGGAATTTACAGGGACTAA
TTAAATGTTTACATTAGGGATAACAAGCCCTGCCTAGGATCAGCCTAATGCCAGGCAAGAATTTCACACTTTAA
GGTTAAAAGCATCAGGGGGTTGTTAACTCTTGACAGGCCTTAAGAGTAATGTGCTATCACAGAGCTCTAAATT
CTTAGGTCTAATAAAACTTATCTTGTCTGGAGAGTTTCCCCTTATCAGGGTAGTATATCAACTTATACTTGACA
TGGAAGTAGCCTGTAGTAAAAGATTTCTATCTCAGTGAGACTTTTAGTCTCTATCTGTAAACAGCTGAGTAAA
ATGGCAAGTGCTTAATTGTTTACTGAATGGGTTAAGCTCCTTGCTGCTATCTGGAATCTAAGAACACTGGAAA
AAGGCTTTAGCTATGTTAGAATACAATCTTAAAAGGCATTTACTATAAGGTAATACTATATAGAGTGCACGTG
GATCTATACACTAGACTAATGTGGGAATGGAAAAATTAATTTTATGGTTTATATAGATTAGGGAGATAATGCC
AGGCCCCCGGGAAGGGAGTTTCCTTGAAACTCTTTGCTTCATGAACCAGCTTCCCAGCTTTCGCTTTGTCAAGCT
GACTCCACCAGAGTCCCTGGCATTCCACCCTTTTTTTTTAATAATTGATAATTTATCTTTATTTCTCAGCCGAAGT
TCCAGGATGTCCCGCTTCGTCGTAGCTACAGATCTCAGGAGCACTCTAAAGATGAGTGGAAACACAATGATTACTAAT
AAAGTAATGCCAAAAATAATAACAAGTAGGATAATATATTGAACCCAATCCAGAGGGTTTAATGCACTTAAGTTGTT
CTTTAAATCTCTAGTCAATTCATCAATGTTCCAAGTGTCCAGATGGCTTTTACTAATATCTGAAATTTTTAACTTTAA
CTGTTCTAAATCATGAGAAATATCAGTATCTTTCCATATACCCTGTAAATGAGTTTTTGTCTTTTCCTAATCAGTGGT
TGTAAGGTAAAGGAGTGACATAAATGTATAGAAAAGAAGCATGACATTTAGTAGCTAACCTGGTCTTGATATTAGCA
ATATCTTGTCCTATTGTCAGCACGACTTCTTTTAGGGCGTTAACCTTTGCTTCCAATTTTTTATCTATAAAATACCAC
TCTGACAAGGCTACAGAGATATTCTTATGCATGTCATTAACAAAATGAGCAGTATGCAGTTGTTGTACTAAGGCTGT
GGTGGATAACGTAAAGGAAGTTAAAATAGCAATTAAAGCAGTAATTTCCAGGATCAAAGCTGCTACAAATCTCTTAG
GTCTAATAAGTTCTTTTAGTCTTTTTAAAGCTTGCAATCCCGAATTCTCAAACTAAGGATCATTTTTTAACTCTACAG
GCAGTAAAACATATCCAGGTCTCTTAAGCAAAATCATAATAGCAGATTCCTTGTCTAAATTAGGTTCTACACAATTA
GTTAATTGACAGGAATTACAATGTATATAAAAAGACTTGTTAAGATTGGTGATCTTAACTCTTAAAATTATCTAACA
ATAAGCATACAGGTAAGGTACACAAGCTTTTACAGCTATAGGGGAGTTGGCCTGAATGGCCAAAAACAGTCTCTGAT
GAAATCTTGTCTAATACTCTGTAGCAGTCTTTCTACCTCACGAGTCAGCTGTTTTAGTTGTCTCCAAGTTGACACTAG
GTCTGCTCTCCTTGTTGTAGTCCATGGCGGAGATAGGAAGAGTTTCTTTATTTTGCTTGTGAGTGACTGATTTTTTTT
TCCTCTGTGCACTCAATACAGCTTTCTGGTCACCACTGTCAGGGCACTCAGGAACTCACAGGTCAGCCTGTCACATGA
ATCATTTTGGGAGCTAGCACGCTCTTGTAGCATTCTGTGGAAAAAACAGAAATATTGCCCCCTTTTCCCCATATTAAG
TATCTGAACAGGATCATACCATGTGTCAATAAGTGGATCCCTTTATCTCATGAGTATGTCTGATTGTAGGGTGCCATA
GACACTTAGAGAGATTTTTGACATCTAAATTTTTAAAAGATATAGATTTAAAGTTCCACAGTACTGTTTTCTTGGGA
ATAATAAAAATCTCAGTAACATGGATAACATTAAATTGTTGATAAAACATCTCAAATGCTTGGCTATAATAGCCAGT
TTTAATTGATTTGGAACACCAAGTAAAAAAATCAACATAGGCAACTATACTTTTAGTTGCTATTTTTATTAGAGCAG
TTGTAAGTAGAAAGATTAAAAAGGTATCAATAGTCACATGTGCATGATTTATTTTTGTAAAATTAAAAAGTATCTAT
TTGTAATAATTGATTAAGTATACGTCCTCAAGAATTAATACCAATGTGTGTAACAGGTAGAAACTGAGGACACTGAG
ATCAAATCTTTACAATATGACATGTACATTAATTAATTTATTTATTTATTTATTTAAATACCAAATTATTATCTCA
AGCATTACTATTTTGATGATGTAAAGAATGAGATTATATGGCATTTATTTTTTTTTATAAGATCTATAATTTACCTG

GGATAGAAATCTAGCGTGGCATTGCCTTAAGAGGTTTTTATCAGGCATTCTTTAACATTTTAAAACACTATCTACAGC
ACATAATTTAATTATCTGTGCTAAAGTCGGCAGAAACTCAAAAGAATAAACATGTGATCCAATTTACATATATTTTT
CTTTCCATAGAAAGGTTGTTTTTAAATACAGTAAATGGGATGCATGTATGTATGAATTTACAAAAGTATGTATAGTA
AGCAATTAGCTTATTTTTAAGATGAATATTAATTTTATTTAAAATGATGGTATGCAATAGGTCAAATATCAATATTT
TGTAATAACTAATAGCTGTTTGGAAAAAAGAATTTTTAATTCTTAATTAGTACCACAGCAGCCTTATATATAATAGG
ATGTTAAAACTTTGAGGTGAAGAAAGATGATTTTTACATTAATGTTTTTTCTTGTCAAAAAATAGCTGTGGACACAT
TTAATAACAATAACTAGAATATGTAGCTAATATTTGATTACCATCAATTACAATCGATATCAATATATTATAGAAGT
TTTCTCTACTTTCTGTTAAAGCCTTTTTAGTTCTCACCAGTTAACTTGAACTTTACTTGATACCACTTAACAAAGGCT
TAGGTTTTCTTGTGGTGAATTTAAGGTGAGGTCTTAGTCAATTAACATCTCTTGGAAGCTTTTTGAAAATAAAATTT
TGAAGTAAATTTGTACCAATTTTTTAATATAACTAAAACCCTAGATATTAAAAAAAGACATTGCCTCTGAATCTTAT
GAAGCAACAACTATTTTCCAGAACTTTAAACTTCTCTTTAGAGACATTAGTTAATAAAATACTTTCCACTTAGGAAA
CAATATATACTGAAAGATTTAAACTATTTTGCTTCTTGTATTGAAGCCTGGACAATGAAAATTTCTTACATAATATA
AGACTATATTAGCCATACTTAGGGGTAAAAACCTTTAATGGTCACCAGTTCACTAGAAGTAAACTCTTTATAAATGG
AAAAAATAGACTTTTAAATCTATAATAATTTTACATGAAGTAGGCAAGTTATTTTGTAATGCCTTTATAAGTTTTAT
AGCCTGATTGACCTTTTATAAATCTTGAATTTAGACTTAATCATGAACAACACTTGGGTAGATCTGAAAAGAAAAAA
TGTTGTTTAGCTAAAAGAAATTCTCCCTGAAGGCAGGGAGGGTGAAGTTTCAAGAACTTTTTTAGGCGCAGTCTGTA
AAACGCAAGAAACTTTGCACAAGCTTCTCTGAGGCTGCTTTAAGTTTTGTATTAACCCAAATGTATATATTTTTTAAT
TTGAGCATCTTGCCCTCCTGCAATGAGGACAGATTCTAAAGGAGCAATTTAACTGTTTGTCCATGCCTCTGATTTTGG
ACACTCTTTCTTAAGATGACTTATACCTTAAACATTTTCTTTTATGACCTTTTATGGTCCTGCAATGCCGCAACCATA
GTCAAATTGATTGTGTGAGGGTCTAATTTTTGTACAAAGATGAATATATCTAGTTAATTTTACTTTGTTTTGTATAG
CTGAATGTCCCAAGTGAGCAGCATTAGCACTCTTATAAGTTGATTATTCTAGTAATAATCTTAAGTATGGGTGAGTT
AAAAGTCTTAAGCTTTTGATTAAACTGCAAACTGCAGTCAATGGGACACTGGCAATTTTAACCAGAATTTTTAAGTT
TTTGTTGCAATATTAGAATATATCTATAACTTTTGATAAGTAAGACCCAATTTTAAACAATTTATTCTCCTTAAAAT
CAATACTAAAAACATCACTACTACGTTACAAAATTTGGCTGTTAATTCATTAATGTATGACAGTTTAATGTTTCATT
AAAGAGACATTATCTTTAAAGATAAGAATTTCATAAAATGTTATCTCAATTTAGAAGTATCTTTAGAGGACTAGTTC
TCTGGGCTGATTTATGCCAGGTGCTTTTGTTTGAAAATGACTCTATCTTTGAGTTACTACTTTTAAGTTAACTTTGTA
ACTAGTGTTACATCTTTTTAATTATACCCAAATAACTTATAAACCAATTCTCTATGACCAAGACATGTTAGATCATAT
TTATACCTTTAAGACCAATCAAAAACCAAATGAAGTGGCTACATGAATCTTATAAATTTAGATCAATTAAAGAGTTT
TACCTTGTAAGATAAAAAAGCAATTTGTTATTTGCTAAACACAATGATCACAAACTTGCAGAGAAAGTTTTAGGAAA
AAACAACTATCAGCTTATTTGCCTTAGAACTAAGAGGTTGCAGATTCCTTTTCTTTAAAAACAGTTTCTAGCAGGGCC
TCTCTTGCAGTGCTTGATTTATGGCTATAGTGAAGGCTCTTTCTCAGCCTCCTTTGTGCAAACTGAGAGTGAATCAGC
AGGTAAAGTTTTAACCATTATTTTTGTTTTATTTTTTCAACATGAAACATAAAACAGTCATTCAGACAACGACACAA
TCATTCAGACAATGAAACAAAAGAGATATACATGAATTGACATTAGACAATACATATAGGGGGTAAAGAATTTCTCC
TGTAGGGGCCAGAGAGAATGAAACAGGGCGTCCCCCGATTTCTCTCTGTCCCTGGGAGAATTCTAAAATCTATTTTCT
TTTATCCCTTCCTTTCTTTTCCTGCTTGTAAGAGACAGGTTGTCAAATCAAGATTAAAATCCAAAATTTTCTAACAGC
TCTGGCAAGCATCCAAACTTCTAATTTATTTAATCTGAGGGCAAATTTTTGAGAAAACACACAATCTCTATACACTAA
AATGTCTCAAATAGCCTAAAAGTTTACCATGCTGAATCTTTTTGAAGCCATCATCAGTCCACACTTAGTCATATATTT
TATAGTCACCTGCAAAAAATGCTATTTTCTGCAACCAACAAAAATACCATTTATGGAATGAATTATGTATGTTTTAG
GAGATTACTTAACTTTCAATAGAGCCTTTTCTACGGATTTTTAGCACAATGTAGGATTGGGAAATTGTAAATAAGGT
AACAGTAAGAAGTGACCTATATTTTGACTTTTAAGTCTCTTTTTATACTTTGACCATAATTTAACCATAACTTTTAAT
TTAGCCTAAAGTATCAGAATTTACAACTTGAAGTTTTTCTTATAACTGGAGCTGTGTCCTATAATGAGCTCCACAGTT
TCTGAACGGGGCCAAGAGACTGCCCCTTTAGTCTTTCGCCTTACCTGCTTCTTAGTGAGCAGAGTCCCATCCTTATGG
GACTTTGATTTACATTCACTTCTCCAGTGCTTGTCACAACACTGGGAGGGACCCAAAGGAATGCTCTTTTTGTCTCCT
GAGAGATTTTTACAATCTTTTCTCAGGTGCCCCGGTTTCTTGCAACCGAAACATGTAATTCCACACGTAGCGCCGAAT
TTTTTCCCTTTCCTTGCTGTGGCGTAAGCTAATTTCTGCATTACAGCCAGTGAGGCATCTACACATGCTTTAATATAA
TCTTGTAGGGAGCCTGTCTTACGAATTGGTCTGATCAGGTCTTGACACAAGGTACTAGCGTTTTCCCAAGCCAATTGT
TTCATTAGTATATCAGTACCTTCCGAGGGAGGAAGCATCCTGGAAACTGCCTCTTCCAATCTAGCTACAAAACTTTCA
TAAGATTCATCATGCTTTTGTCTAATTCCTGAAAGGACTGTGGATTTAGCCCCAGGTACAGGTAAACTCCGCCAGCTA
GATATAGCTATCTGATTTATTCTCTCCAACACTGCCTTAGGAATATTCAGTTGCGCTGCTGGTCTAGCATAGTCTTTT
TGCCCAAGAAGCATAGGGACTGTGGGTCTAGCTCCATTTCTTGATGAAGATCTTTCCTGAGCTAACTTTCTGGCCCTT
TCTTCATATTCTGTTCTCCAGAGAATGAAATCTCCCGGAGCAAGAGTAGCTTTCGCGGTTTGGTACCAATCATAAGGG
GTCAGCCACTGACTAGCTACTGCATCCAGGACCTGTAAAGTATATGGTGCAGTAGGGCCCAGCGTTTTTACTGCTGAC
TGTAATTCCTTAAGAGTTTTTAACGACAAAGGTTCCCATTGTGGCTCACTCCCTTCTTCACAGATTACCTGGCAAGTG
AGTGTAAAGTCTCCTTGCCTTTTTGCCTCTGCCACAGCAGCATCAAAGCCTACCGGTGGCAAAAGTCTACGTGGCTTT
GGCACTGGTCTGCGGGCGGCTGGTGTTATAATTAAAGACTCCTCCTCACTGCTATCTGTGTGCTTACTAAAACGATCC

TTTCTTCCCTTAATTACACACTTTGCCATTCCCTTAGTCCTTTCCCATTCATCATCATTTTCACTGTCAGTGATCTCTG
ATTCACTTTTTTCTAGCTTTATCCCTTTTTTTCTTGAACAATACGATTTCTTTTTACCTGTATTGGGTTTCACTTTCT
GATCATCAGAGAGCTCTGAAGGAGCTTCCTCTGCAAGGAATTCTAGGTCTGTTTTCTCAGTCAATAAATCCCTCATCT
GTTGCCACAATATAAAAGCACTGTCAGGAACATTCCTGGATAAATTTTCCTCGTGAAACTTCCTCATATCCTTTCCTA
CTCGCTTCCAATCTTCTAGTGTCAAGCTTCCCTCCTCTGGAAACCAGGGACTGATTTTTTGCACGAAATCATAAAATT
CTTGTAAAGATTTATCTGAGATTTTTAGGCCTCTTCCAGACAGCATGTGTTTAAACACTGACAGGAACAATTTTTTCC
CTTTTGATTCTGATTGCCCCATGATAAATACTTACCCGTCTTTATAACGAACACTCCAGTCCGCCTGCCTGCAGCGCA
GTCCCACGGGGTCCTATCTTTGCCTGAGAAAATAAAGAATAGAAAGAAAAAAAAGGAAAGAAAAGAAAAGACTAATC
TGCCCAGTTGTCCCTGTTC**GAAGGCGCCAACTGCCACAGACAATCTGGGTCCCTGGTCTGTGAAGAACGGGATTT**
CTTAGGCAGATGGACAAAGATTCCGGATGAAAATGACAGACAGATCACACGAGAAATGTGTTGAATCTGAATG
TATTGTTCTGGTTGAGCTTCAGACTTATATAACAACGAATTATCAGAGGATACAAATCACAAAAAGACAAGAT
ACACTGAAATTCACCAGTTACAGCAGAAAGGAATTTACAGGGACTAATTAAATGTTTACATTAGGGATAACAA
GCCCTGCCTAGGATCAGCCTAATGCCAGGCAAGAATTTCACACTTTAAGGTTAAAAGCATCAGGGGGTTGTTAA
CTCTTGACAGGCCTTAAGAGTAATGTGCTATCACAGAGCTCTAAATTCTTAGGTCTAATAAAACTTATCTTGTC
TGGAGAGTTTCCCCTTATCAGGGTAGTATATCAACTTATACTTGACATGGAAGTAGCCTGTAGTAAAAGATTT
CTATCTCAGTGAGACTTTTAGTCTCTATCTGTAAACAGCTGAGTAAAATGGCAAGTGCTTAATTGTTTACTGA
ATGGGTTAAGCTCCTTGCTGCTATCTGGAATCTAAGAACACTGGAAAAAGGCTTTAGCTATGTTAGAATACAA
TCTTAAAAGGCATTTACTATAAGGTAATACTATATAGAGTGCACGTGGATCTATACACTAGACTAATGTGGGA
ATGGAAAAATTAATTTTATGGTTTATATAGATTAGGGAGATAATGCCAGGCCCCCGGGAAGGGAGTTTCCTTG
AAACTCTTTGCTTCATGAACCAGCTTCCCAGCTTTCGCTTTGTCAAGCTGACTCCACCAGAGTCCCTGGCAACAG
GGATGACTTCTGTGACCCAAAATTCAAGCACAATTCACTAGCTGTA.
